# Autotoxin Rg_1_ Induces Degradation of Root Cell Walls and Aggravates Root Rot by Modifying the Rhizospheric Microbiome

**DOI:** 10.1128/spectrum.01679-21

**Published:** 2021-12-15

**Authors:** Yanguo Xu, Min Yang, Rong Yin, Luotao Wang, Lifen Luo, Bianxian Zi, Haijiao Liu, Huichuan Huang, Yixiang Liu, Xiahong He, Shusheng Zhu

**Affiliations:** a State Key Laboratory for Conservation and Utilization of Bio-Resources in Yunnan, Yunnan Agricultural University, Kunming, China; b Key Laboratory for Agro-Biodiversity and Pest Control of Ministry of Education, Yunnan Agricultural University, Kunming, China; c Yunnan Institute of Tropical Crops, Jinghong, China; USDA—San Joaquin Valley Agricultural Sciences Center

**Keywords:** autotoxicity, soil-borne pathogen, microbiome, rhizodeposits

## Abstract

Management of crop root rot disease is one of the key factors in ensuring sustainable development in agricultural production. The accumulation of autotoxins and pathogens in soil has been reported as a primary driver of root rot diseases; however, less is known about the correlation of plants, their associated pathogens and microbiome mediated by autotoxins as well as the contributions autotoxins make to the occurrence of root rot disease. Here, we integrated metabolomic, transcriptomic, and rhizosphere microbiome analyses to identify the root cell wall degradants cellobiose and d-galacturonic acid as being induced by the autotoxic ginsenoside Rg_1_ of *Panax notoginseng*, and we found that exogenous cellobiose and d-galacturonic acid in addition to Rg_1_ could aggravate root rot disease by modifying the rhizosphere microbiome. Microorganisms that correlated positively with root rot disease were enriched and those that correlated negatively were suppressed by exogenous cellobiose, d-galacturonic acid, and Rg_1_. In particular, they promoted the growth and infection of the soilborne pathogen Ilyonectria destructans by upregulating pathogenicity-related genes. Cellobiose showed the highest ability to modify the microbiome and enhance pathogenicity, followed by Rg_1_ and then d-galacturonic acid. Collectively, autotoxins damaged root systems to release a series of cell wall degradants, some of which modified the rhizosphere microbiome so that the host plant became more susceptible to root rot disease.

**IMPORTANCE** The accumulation of autotoxins and pathogens in soil has been reported as a primary driver of root rot disease and one of the key factors limiting sustainable development in agricultural production. However, less is known about the correlation of plants, their associated pathogens, and the microbiome mediated by autotoxins, as well as the contributions autotoxins make to the occurrence of root rot disease. In our study, we found that autotoxins can damage root systems, thus releasing a series of cell wall degradants, and both autotoxins and the cell wall degradants they induce could aggravate root rot disease by reassembling the rhizosphere microbiome, resulting in the enrichment of pathogens and microorganisms positively related to the disease but the suppression of beneficial microorganisms. Deciphering this mechanism among plants, their associated pathogens, and the microbiome mediated by autotoxins will advance our fundamental knowledge of and ability to degrade autotoxins or employ microbiome to alleviate root rot disease in agricultural systems.

## INTRODUCTION

The root systems of plants are responsible for the uptake of water and mineral nutrients from soil (1, 2). Therefore, root rot is a problem for crop production. Monocots, dicots, cereals, legumes, fruit trees, and tubers have all suffered from root rot diseases (3). Due to the involvement of more than one pathogen, the disease is commonly referred to as a root rot complex. The symptoms of root rot include soft, water-soaked, dark brown to black lesions on the roots, yellow color and wilting of leaves, causing stunted plant growth and reduced yield. Cultural, physical, biological, and chemical control methods have been used as management strategies to control root rot diseases (4). However, these control methods have only been partially successful because the pathogens and other factors are complicated. There is a critical need to understand the mechanisms of root rot occurrence and develop suitable and sustainable measures to control root rot disease.

The buildup of detrimental soilborne pathogens, such as fungi, bacteria, oomycetes, and nematodes, is an important factor leading to root rot diseases (5–8). Furthermore, the buildup of soilborne pathogens is often accompanied by the suppression of antagonistic microorganisms (9–11). Because of the complexity of the soil environment, the occurrence of root rot disease is regulated not only by soil microorganisms but also abiotic factors. Rhizodeposits (e.g., exudates, border cells, and mucilage) of plants have been reported to shape the rhizosphere microbiome and then affect the severity of root rot disease (12). Autotoxins, a kind of rhizodeposit, are released from plants into the environment to inhibit the activity of the same plant species (13, 14); moreover, some autotoxins promote the growth and pathogenicity of pathogens (15, 16). Therefore, some autotoxins show synergistic effects with soilborne pathogens to aggravate root rot. In addition, autotoxins can affect the interactions between plants and the rhizosphere microbiome. A series of studies showed that many autotoxic phenolic acids could modify the rhizosphere microbiome and affect the disease process (17, 18). On the host side, soil abiotic stress was reported to change the metabolites of plants and then affect the release of rhizodeposits, which finally modified the rhizosphere microbiome (19, 20). Some autotoxins have been reported to induce changes in roots at the transcriptional level (21, 22). Whether autotoxins induce changes in rhizodeposits is still unknown. In addition, how these autotoxin-induced rhizodeposits affect root rot disease through modifying the composition and function of the soil microbiome remains to be determined.

*Panax notoginseng* (Burk.) F. H. Chen, a member of the Araliaceae family, is suffering from severe root rot diseases because of the buildup of soilborne pathogens and accumulation of autotoxic ginsenosides (23, 24). Here, we used P. notoginseng as a model plant to (i) identify modified metabolites in plant roots exposed to the autotoxic ginsenoside Rg_1_, (ii) test the effects of exogenous Rg_1_ and two Rg_1_-induced root degradants (cellobiose and d-galacturonic acid) in soil on root rot disease, (iii) unravel the effects of Rg_1_, cellobiose, and d-galacturonic acid on the rhizosphere microbiome, and (iv) identify the effects of modified culturable fungi and bacteria on plant health *in vitro* and *in planta*. The underlying mechanisms of the effects of Rg_1_, cellobiose, and d-galacturonic acid on the growth and pathogenicity of soilborne pathogens were also explored by transcriptional analyses.

## RESULTS

### Rg_1_ disrupted root cells to release cell wall degradants.

**FIG 1:**
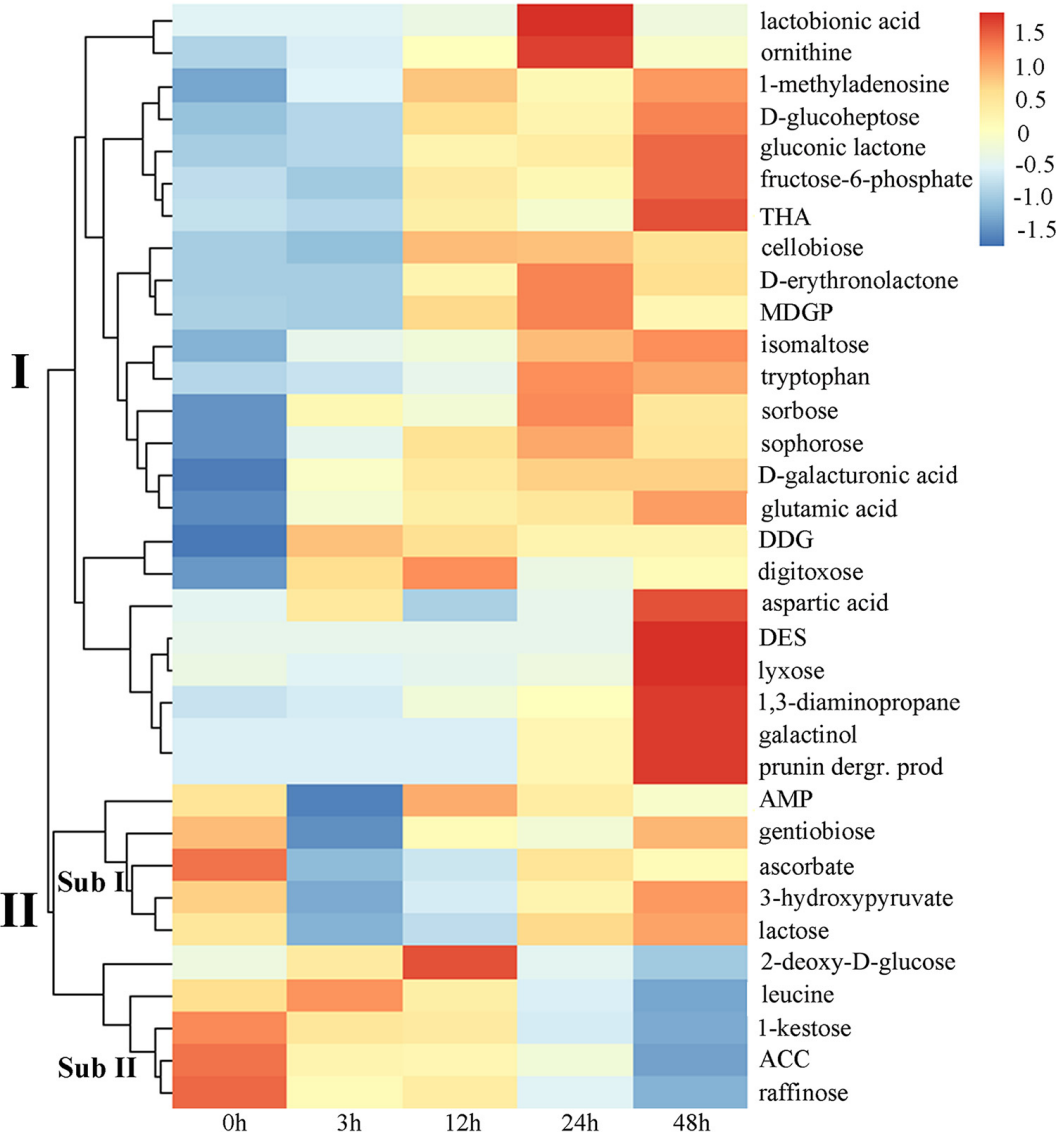
Hierarchical clustering of differentially expressed metabolites in roots after exposure to Rg_1_ for different times. The metabolite concentrations at each time point (six biological replicates) were normalized by a Z score transformation and are represented by the Z scale. THA, threo-β-hydroxy aspartate; MDGP, methyl-β-d-galactopyranoside; DDG, 2-deoxy-d-galactose; DES, d-erythro-sphingosine; AMP, adenosine 5-monophosphate; ACC, 1-aminocyclopropane carboxylic acid. Detailed descriptions of these metabolites are given in Table S2.

A total of 524 metabolites across all samples were detected. An excellent separation according to 0, 3, 12, 24, and 48 h of treatment with the autotoxin Rg_1_ was achieved by an orthogonal partial least-squares discriminant analysis (OPLS-DA) score plot (Fig. S1A in the supplemental material). Overall, 34 significantly modified metabolites were found in the roots after treatment with Rg_1_ for different lengths of time (Table S2). Hierarchical cluster analysis (HCA) grouped the significantly changed metabolites into two major clusters (Fig. 1). Metabolites in cluster I, including cellulose degradants (cellobiose, sophorose, and gluconic lactone), pectin degradants (d-galacturonic acid), and hemicellulase degradants (lyxose and methyl-beta-d-galactopyranoside), were upregulated in roots after exposure to Rg_1_. Cluster II could be divided into two subclusters. Metabolites in subcluster I, including ascorbate-glutathione (AsA-GSH) cycle-related metabolites like gentiobiose, ascorbate, and 3-hydroxypyruvate, were significantly downregulated at 3 h after exposure to Rg_1_ and then returned to normal levels. Metabolites in subcluster II were significantly downregulated from 3 to 48 h after exposure to Rg_1_. The metabolite-profiling data were validated by gas chromatography-time of flight mass spectrometry (GC-TOF MS) combined with 13 randomly selected standard compounds (Fig. S1B). Among the metabolites in cluster I, the cellulose degradant cellobiose and the pectin degradant d-galacturonic acid were significantly upregulated at all treatment times (Fig. S1B, Table S2). Therefore, we chose these two cell wall degradants for further study.

### Autotoxin Rg_1_ and cell wall degradants aggravated root rot disease.

**FIG 2:**
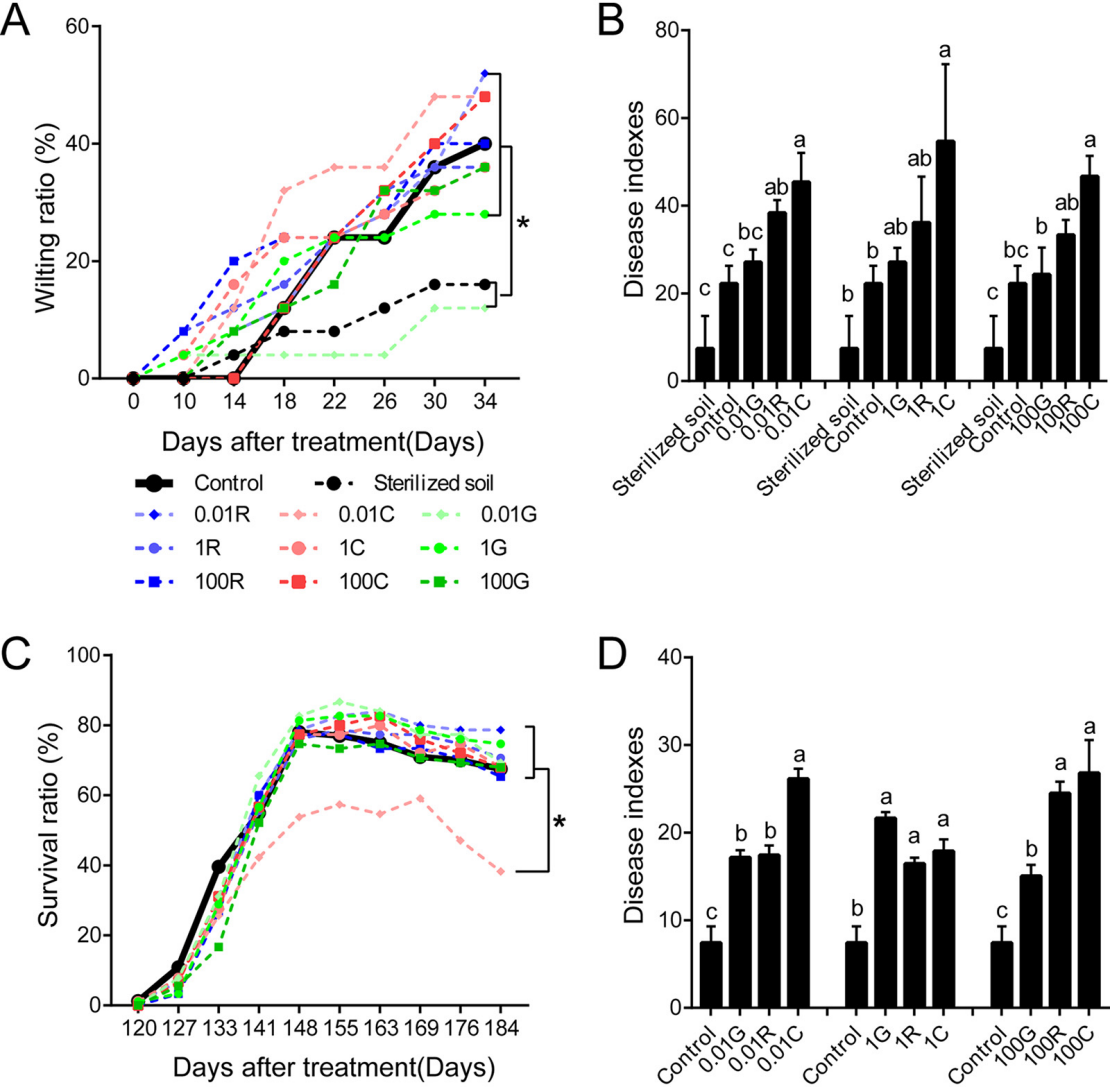
Effects of Rg_1_, cellobiose, and d-galacturonic acid on root rot of *Panax notoginseng*. (A and B) Progress of wilting symptoms (A) and root rot disease index values of P. notoginseng after exogenous addition of different concentrations of Rg_1_, cellobiose, or d-galacturonic acid to conditioned soil. (C and D) Seedling survival rates (C) and root rot disease index values (D) of P. notoginseng after exogenous addition of Rg_1_, cellobiose, or d-galacturonic acid at different concentrations to natural pine forest soil. Blue, red, and green dotted lines represent Rg_1_, cellobiose, and d-galacturonic acid treatments, respectively. R, Rg_1_; C, cellobiose; G, d-galacturonic acid. The concentrations of these three substances were 0.01, 1.0, and 100 μg ml^−1^. Bold black solid lines represent control, and black dotted lines represent conditioned soil sterilized at 121°C for 30 min. Experiments were repeated three times; there were five pots for each treatment with six seedlings per pot in conditioned-soil experiments and 15 pots for each treatment with five seedlings per pot in natural-soil experiments. Error bars represent the standard errors of the means, and asterisks and different lowercase letters indicate significant differences between treatments detected by one-way ANOVA and Duncan’s multiple-range test (*P < *0.05).

Rg_1_ and the cell wall degradants cellobiose and d-galacturonic acid were exogenously added into conditioned soil, which was prepared by blending 5% soil in which P. notoginseng had been cultivated continuously for 3 years into 95% sterilized natural soil (without P. notoginseng cultivation history), to observe their effects on root rot disease. Compared with the growth of seedlings in conditioned soil alone, the wilting symptoms of seedlings progressed quickly in conditioned soil amended with exogenous Rg_1_ and the cell wall degradants cellobiose and d-galacturonic acid (Fig. 2A). The ratio of wilting seedlings decreased significantly when conditioned soil was sterilized (Fig. 2A). The severity of root rot disease was significantly increased when conditioned soil was amended with Rg_1_ at 0.01 μg ml^−1^ and cellobiose at 0.01, 1.0, or 100 μg ml^−1^ for 34 days (Fig. 2B). The severity of root rot in d-galacturonic acid treatments also increased, but it did not differ significantly from the severity in the control (Fig. 2B). Furthermore, these three substances were exogenously added into natural soil to observe their effects on root rot disease. Wilting symptoms progressed more slowly in natural soil than in conditioned soil, and the survival ratios exhibited no significant differences in exogenous-addition treatments compared with the control, except for the cellobiose treatment at 0.01 μg ml^−1^ from day 141 to day 184 (Fig. 2C). However, at the end of the experiment (day 184), the severity of root rot disease was significantly increased in all treatments amended with Rg_1_, cellobiose, or d-galacturonic acid at a concentration of 0.01, 1.0, or 100 μg ml^−1^ (Fig. 2D). Notably, cellobiose showed the strongest ability to aggravate root rot disease, followed by Rg_1_ and then d-galacturonic acid, in both conditioned soil and natural soil (Fig. 2B and D). Ilyonectria destructans was frequently isolated from roots with typical rot symptoms and was identified as the pathogen by Koch’s postulates.

### Rg_1_, cellobiose, and d-galacturonic acid modified the rhizosphere microbiome.

**FIG 3:**
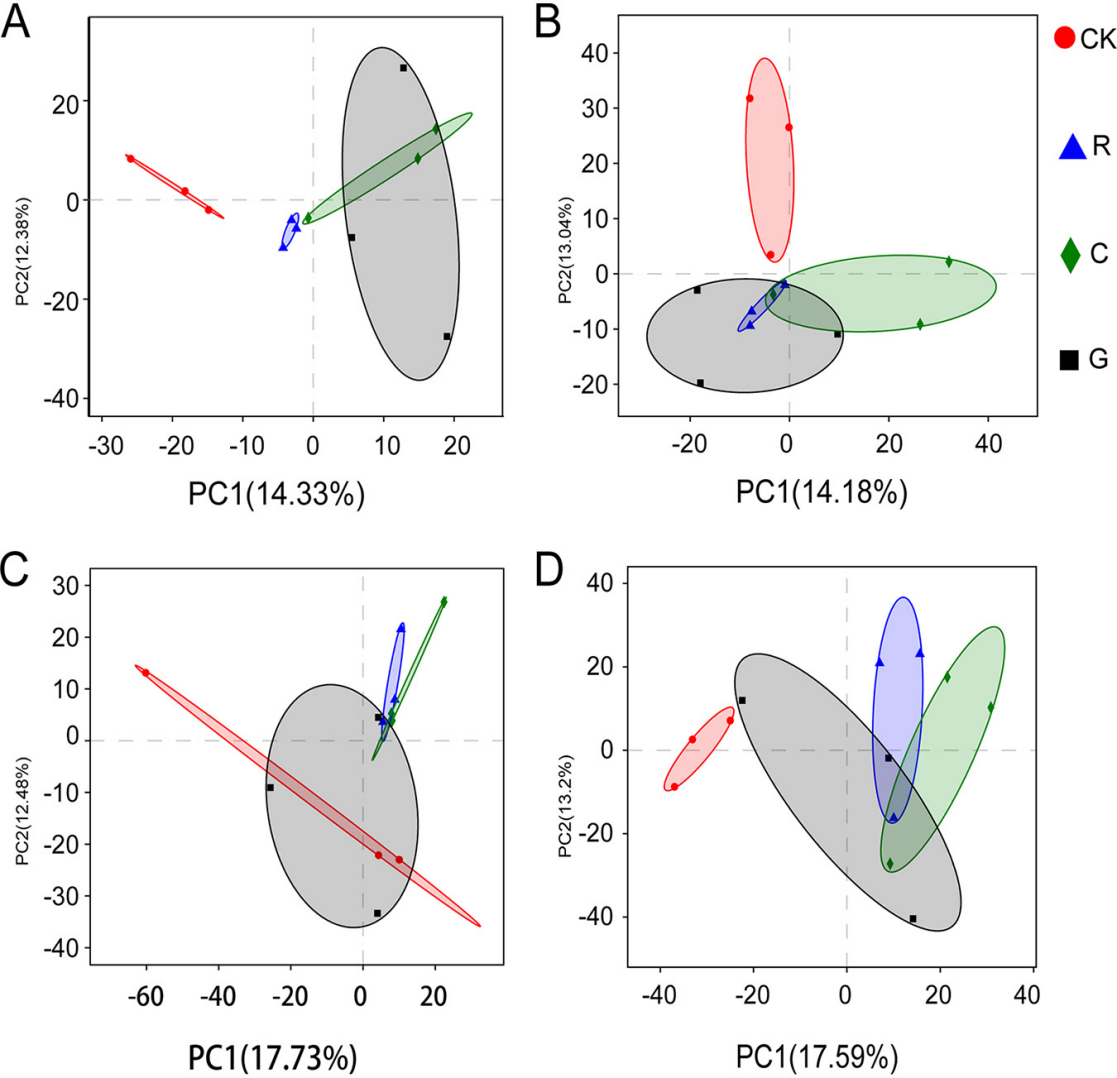
Principal-component analyses (PCA) of operational taxonomy units (OTUs) in the rhizosphere microbiome of *Panax notoginseng* after exogenous addition of Rg_1_, cellobiose, or d-galacturonic acid in conditioned soil and natural soil. (A and B) PCA of fungal (A) and bacterial (B) communities in conditioned soil. (C and D) PCA of fungal (C) and bacterial (D) communities in natural soil. PCA was conducted after the OTU abundances were standardized. Numbers on the horizontal and vertical axes represent the interpretation of the respective principal components. CK, sterilized deionized water; R, Rg_1_; C, cellobiose; G, d-galacturonic acid.

Principal-component analysis (PCA) of operational taxonomic units (OTUs) showed that the fungal (Fig. 3A) and bacterial (Fig. 3B) communities were both distinct from those in the control treatment when Rg_1_, cellobiose, and d-galacturonic acid were exogenously added into the conditioned soil. In natural soil, treatments with exogenous Rg_1_ and cellobiose, but not d-galacturonic acid, had fungal communities that were distinct from that in the control (Fig. 3C). The bacterial communities could be distinguished from that of the control treatment after the addition of these three substances (Fig. 3D). Additionally, the number of taxa at the genus level (Fig. S2A) and the alpha diversity of OTUs in natural soil (Fig. S2B) were higher than in conditioned soil.

**FIG 4:**
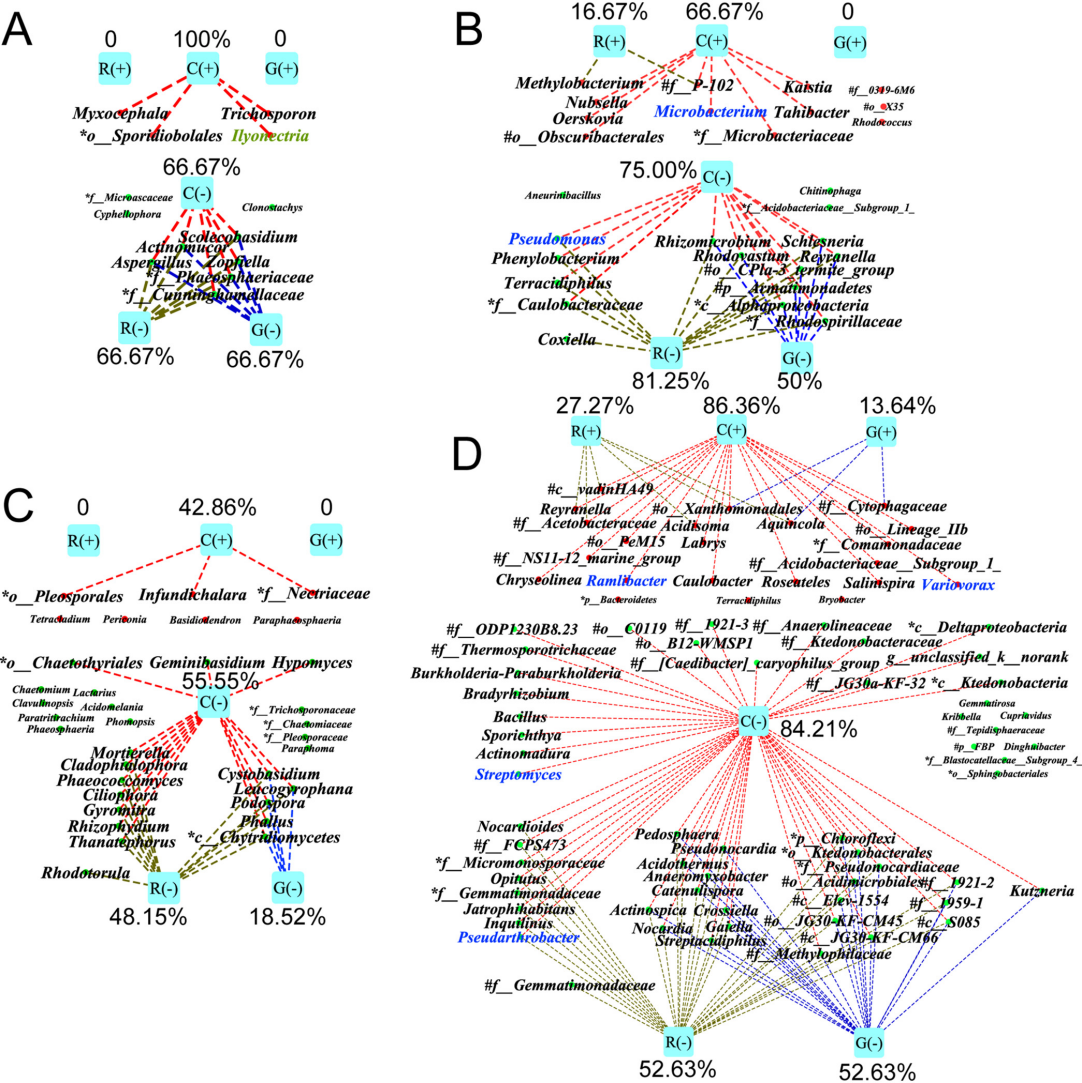
Effects of Rg_1_, cellobiose, and d-galacturonic acid on rhizosphere microbiome of *Panax notoginseng* at the genus level. (A and B) Root rot disease-correlated fungi (A) and bacteria (B) in conditioned soil. (C and D) Root rot disease-correlated fungi (C) and bacteria (D) in natural soil. The red and green dots represent genera that were significantly positively and negatively correlated with root rot disease, respectively; the red, green, and blue dotted lines indicate genera significantly enriched or suppressed by the exogenous addition of cellobiose, Rg_1_, or d-galacturonic acid, respectively. R(+), C(+), and G(+) represent enrichment of abundance by Rg_1_, cellobiose, or d-galacturonic acid, respectively; R(−), C(−), and G(−) represent suppression of abundance by Rg_1_, cellobiose, or d-galacturonic acid, respectively. Correlation analysis was performed in R with Pearson’s correlation coefficient (| *r* | > 0.6, *P* < 0.05). The significance of changes in relative abundances of genera was detected by one-way ANOVA and Duncan’s multiple-range test (*P < *0.05).

In conditioned soil, the relative abundances of 4 genera of fungi and 12 genera of bacteria were significantly positively correlated with the severity of root rot disease, while those of 9 genera of fungi and 16 genera of bacteria were significantly negatively correlated (Fig. 4A and B). Importantly, the relative abundance of *Ilyonectria* was significantly positively correlated with root rot disease (*r *= 0.79; *P = *0.002). Exogenous Rg_1_, cellobiose, and d-galacturonic acid showed different abilities to regulate these disease-related microorganisms. Cellobiose significantly enriched 100% of fungi and 66.67% of bacteria that were positively correlated with the disease, and it suppressed 66.67% of fungi and 75% of bacteria that were negatively correlated with the disease (Fig. 4A and B). Rg_1_ significantly enriched 0 and 16.67% of fungi and bacteria, respectively, that were positively correlated with the disease, but it suppressed 66.67% and 81.25% of negatively correlated fungi and bacteria, respectively (Fig. 4A and B). d-Galacturonic acid had no significant effects on positively correlated fungi and bacteria, but it suppressed 66.67% and 50% of negatively correlated fungi and bacteria, respectively (Fig. 4A and B).

In natural soil, 7 genera of fungi and 22 genera of bacteria were significantly positively correlated with root rot disease, while 27 genera of fungi and 57 genera of bacteria were significantly negatively correlated with root rot disease (Fig. 4C and D). Consistently, cellobiose showed the strongest ability to modify disease-correlated microorganisms, followed by Rg_1_ and then d-galacturonic acid (Fig. 4C and D).

### Bacteria modified by Rg_1_, cellobiose, and d-galacturonic acid affected root rot disease.

**FIG 5:**
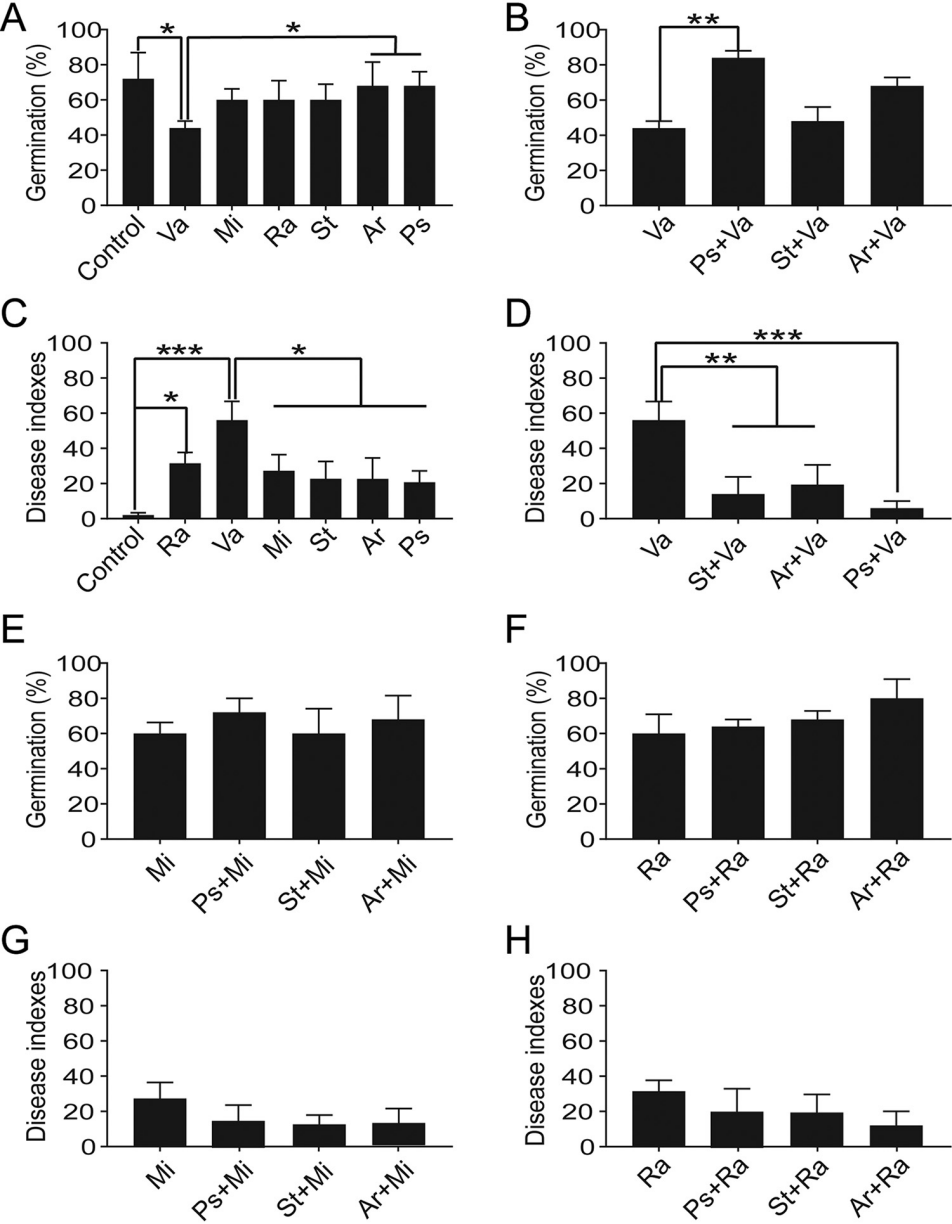
Effects of bacteria positively and negatively correlated with root rot disease on seedling germination and root rot disease of Panax notoginseng. (A) Effects of these six isolates on seedling germination of P. notoginseng. (B) Alleviation by Pseudomonas, *Streptomyces*, and *Pseudarthrobacter* isolates of the inhibition of germination of P. notoginseng caused by *Variovorax* isolate inoculation. (C) Effects of these six isolates on the root rot disease of P. notoginseng. (D) Alleviation by Pseudomonas, *Streptomyces*, and *Pseudarthrobacter* isolates of the severity of root rot of P. notoginseng caused by *Variovorax* isolate inoculation. (E and F) Alleviation by Pseudomonas, *Streptomyces*, and *Pseudarthrobacter* isolates of the inhibition of germination caused by *Microbacterium* (E) and *Ramlibacter* (F) isolates. (G and H) Alleviation by Pseudomonas, *Streptomyces*, and *Pseudarthrobacter* isolates of the severity of root rot caused by *Microbacterium* (G) and *Ramlibacter* (H) isolates. Va, *Variovorax*; Mi, *Microbacterium*; Ra, *Ramlibacter*; Ps, Pseudomonas; St, *Streptomyces*; Ar, *Pseudarthrobacter*. The data represent the mean values from five repeated pots. Error bars represent the standard errors of the means. The significance of differences between treatments was detected by one-way ANOVA followed by Duncan’s multiple-range test. *, *P < *0.05; **, *P < *0.01; ***, *P < *0.001.

A total of 279 bacterial isolates belonging to 38 genera were isolated from the conditioned and/or natural rhizosphere soil (Table S3). Inoculation with an isolate from the positively disease-correlated genus *Variovorax* significantly decreased the germination rate of seedlings compared with the germination rate in the control, as did inoculation with Pseudomonas and *Pseudarthrobacter* isolates (Fig. 5A). Inoculation with pairs of isolates revealed that the Pseudomonas isolate significantly alleviated the inhibitory effect of the *Variovorax* isolate on the germination rate of seedlings (Fig. 5B). Additionally, the *Streptomyces* and *Pseudarthrobacter* isolates alleviated this effect, but these changes were not significant (Fig. 5B). Inoculation of the isolate from the positively disease-correlated *Variovorax* genus aggravated the severity of root rot compared with the severity in other treatments (Fig. 5C). Inoculation with pairs of isolates revealed that the Pseudomonas, *Streptomyces*, and *Pseudarthrobacter* isolates could significantly alleviate the severity of root rot disease caused by inoculation with the *Variovorax* isolate (Fig. 5D). Similarly, the addition of isolates from the positively disease-correlated *Microbacterium* and *Ramlibacter* genera inhibited germination and aggravated root rot of P. notoginseng compared with the isolates from the negatively disease-correlated Pseudomonas, *Streptomyces*, and *Pseudarthrobacter* genera, although these effects were not significant (Fig. 5A and C). Alleviative effects were also observed when isolates of the *Microbacterium* and *Ramlibacter* genera were inoculated into soil in combination with isolates of the Pseudomonas, *Streptomyces*, and *Pseudarthrobacter* genera (Fig. 5E to H).

The application of Koch’s postulates to these six disease-correlated bacterial isolates revealed that they were not pathogens of P. notoginseng (Fig. S3A). Furthermore, isolates from the negatively disease-correlated genera Pseudomonas and *Streptomyces* showed obviously antagonistic activities against I. destructans (Fig. S3B). The *Pseudarthrobacter* isolate and the isolates of the positively disease-correlated *Ramlibacter* and *Variovorax* genera showed slight antagonistic activities against I. destructans (Fig. S3B). Among these six bacterial isolates, the Pseudomonas isolate showed antagonistic activities against the *Ramlibacter*, *Microbacterium*, *Pseudarthrobacter*, and *Streptomyces* isolates (Fig. S3C).

### Rg_1_, cellobiose, and d-galacturonic acid promoted the growth and infection of *I. destructans* to aggravate root rot.

**FIG 6:**
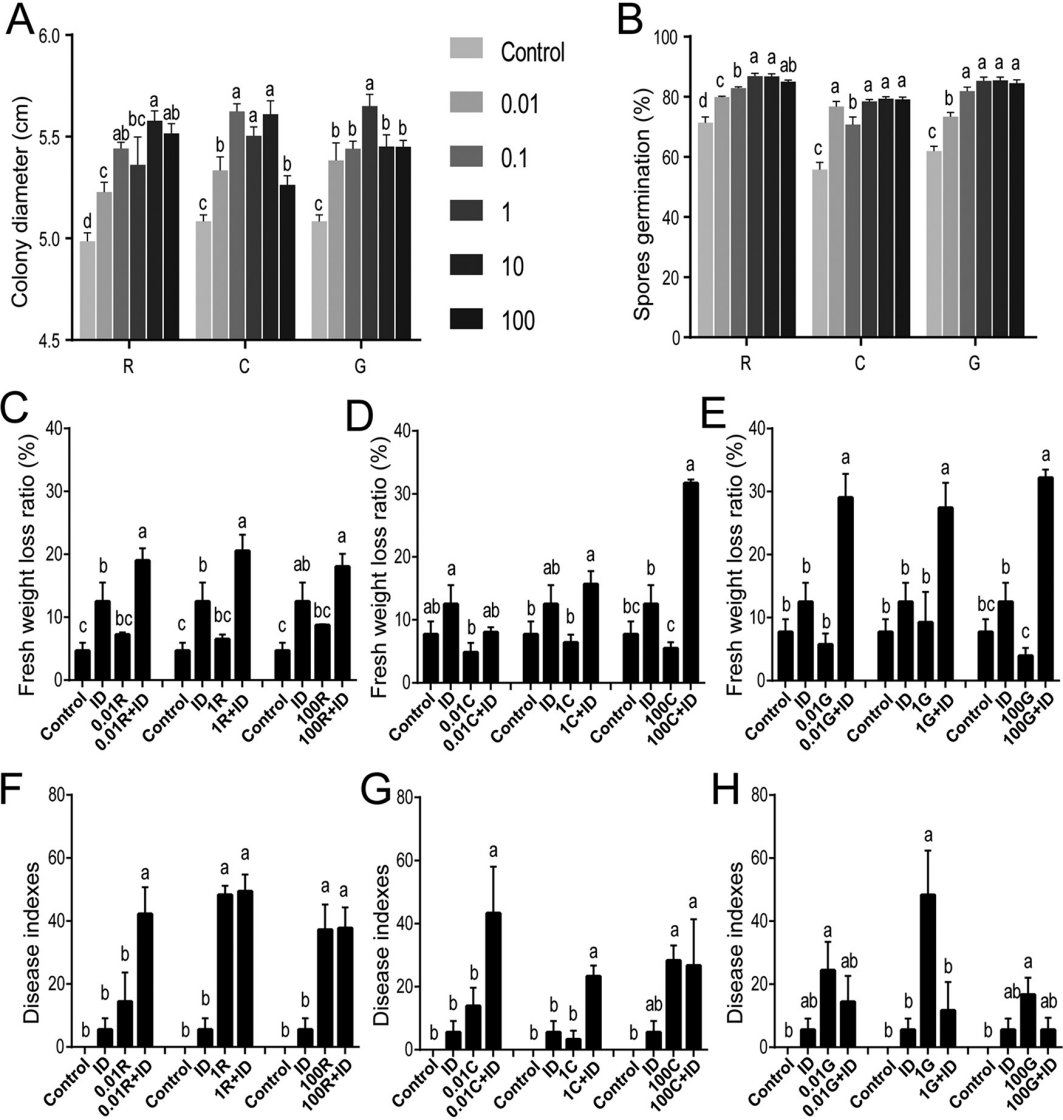
Effects of Rg_1_, cellobiose, and d-galacturonic acid on the growth and pathogenicity of *Ilyonectria destructans*. (A and B) Effects of Rg_1_, cellobiose, and d-galacturonic acid on hyphal growth (A) and conidiospore germination (B) of I. destructans on PDA medium. (C to E) Effects of Rg_1_ (C), cellobiose (D), and d-galacturonic acid (E) on infection by I. destructans under hydroponic conditions. (F to H) Effects of Rg_1_ (F), cellobiose (G), and d-galacturonic acid (H) on the severity of root rot disease caused by I. destructans, in sterilized soil. R, Rg_1_; C, cellobiose; G, d-galacturonic acid; ID, 10^3^ ml^−1^ conidiospores of I. destructans; 0.01, 1, and 100, concentrations (μg ml^−1^) of these three substances. Error bars represent the standard errors of the means, and different lowercase letters indicate significant differences between treatments detected by one-way ANOVA and Duncan’s multiple-range test (*P < *0.05).

The root rot pathogen I. destructans made good use of Rg_1_, cellobiose, and d-galacturonic acid as the sole carbon sources for growth compared with its use of glucose, xylan, pectin, cellulose, and gum guar (Fig. S4A). When Rg_1_, cellobiose, and d-galacturonic acid were added to potato dextrose agar (PDA) at concentrations from 0.01 to 100 μg ml^−1^, colony growth and conidiospore germination were significantly enhanced (Fig. 6A and B).

The effects of Rg_1_, cellobiose, and d-galacturonic acid on the pathogenicity of I. destructans were further tested under hydroponic and sterilized-soil conditions. Under hydroponic conditions, Rg_1_, cellobiose, and d-galacturonic acid each had no significant effect on plant growth compared with the control treatment, but the degree of fresh weight loss when any of them was added simultaneously with I. destructans was aggravated in comparison with the results with I. destructans alone, as well as in comparison with the control (Fig. 6C to E). Moreover, Rg_1_ at 0.01 and 1.0 μg ml^−1^ (Fig. 6C), cellobiose at 100.0 μg ml^−1^ (Fig. 6D), and d-galacturonic acid at 0.01, 1.0, and 100.0 μg ml^−1^ (Fig. 6E) significantly stimulated the pathogenicity of I. destructans. Additionally, Rg_1_ added to sterilized soil alone at 1.0 and 100.0 μg ml^−1^, as well as cellobiose at 100 μg ml^−1^ and d-galacturonic acid at 0.1 to 100 μg ml^−1^, led to a significant increase in root rot symptoms compared with the root rot in the control treatment (Fig. 6F to H). Rg_1_ added to soil at 0.01 μg ml^−1^ and cellobiose at 0.01 and 1.0 μg ml^−1^ significantly enhanced the pathogenicity of I. destructans, resulting in more severe root rot disease compared to the severity with I. destructans inoculation alone (Fig. 6F and G). However, the combination of d-galacturonic acid and I. destructans did not significantly aggravate the severity of root rot disease (Fig. 6H).

**FIG 7:**
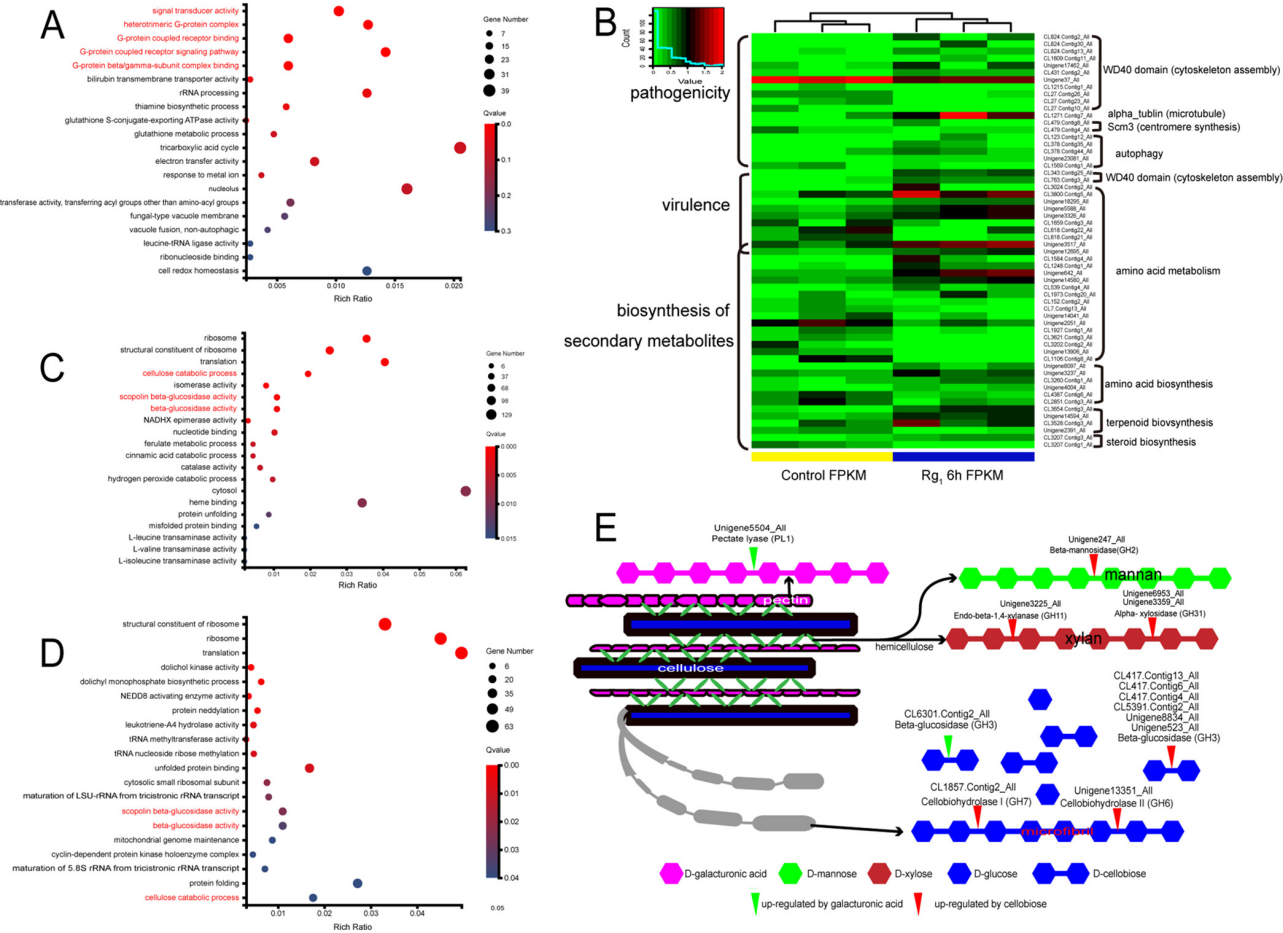
Effects of Rg_1_, cellobiose, and d-galacturonic acid on the gene transcription of Ilyonectria destructans. (A) Gene Ontology (GO) enrichment analysis of Rg_1_ compared with the control (without Rg_1_). (B) DEGs associated with pathogenicity, virulence, and biosynthesis of secondary metabolites between Rg_1_ and control treatments. (C and D) GO enrichment analysis of cellobiose (C) and d-galacturonic acid (D) compared with glucose as the only carbon source. (E) Upregulation of CAZymes genes involved in plant cell wall decomposition caused by cellobiose or d-galacturonic acid.

Transcriptome analysis of I. destructans showed that pathways associated with G protein were significantly enriched after exposure to Rg_1_ at 1.0 μg ml^−1^ for 6 h (Fig. 7A). Among 30 enriched genes, 25 were related to the G protein α I subunit (Table S4). Further analysis demonstrated that some of the differentially expressed genes (DEGs) associated with pathogenicity and virulence, as well as fungal toxin synthesis, were significantly upregulated by Rg_1_ (Fig. 7B). When I. destructans was grown on medium containing cellobiose or d-galacturonic acid as the sole carbon source for 12 h, the pathways involved in carbohydrate-active enzymes (CAZymes) were significantly enriched compared with their expression levels in the positive control (where glucose was the sole carbon source) (Fig. 7C and D). In the negative control (without carbon), however, pathways associated with autophagy and gluconeogenesis were significantly enriched compared with their expression levels when cellobiose (Fig. S4B) and d-galacturonic acid (Fig. S4B) were used as carbon sources, as well as in comparison with the positive control (Fig. S4B). Further analysis showed that there were 6,208 DEGs in the cellobiose treatment and 3,163 DEGs in the d-galacturonic acid treatment (Fig. S4C) compared with both positive and negative controls. Among these DEGs, genes belonging to CAZymes that are involved in cell wall decomposition, including six beta-glucosidase genes (GH1/GH3), two alpha-xylosidase genes (GH31), one beta-mannosidase gene (GH2), one endo-beta-1,4-xylanase gene (GH11), and two cellobiohydrolase genes (GH6 and GH7), were significantly upregulated by cellobiose (Fig. 7E). Among these CAZymes, two cellobiohydrolase genes, Unigene13351_All (GH6) and CL1857.Contig3_All (GH7), were selected to verify the transcriptome profile with real-time quantitative PCR (RT-qPCR). The data confirmed that GH6 and GH7 were upregulated 160- and 187-fold compared with their expression levels in the negative control after induction by cellobiose for 12 h, while these two genes were only upregulated 4- and 12-fold after induction by glucose (Fig. S4D). Transcriptome analysis revealed that one beta-glucosidase gene (GH3) was significantly upregulated by d-galacturonic acid (Fig. 7E). RT-qPCR further confirmed that the pectate lyase gene (PL-1), Unigene5504_All, was also upregulated 31-fold after induction by d-galacturonic acid for 12 h compared with its expression level in the negative control, while it was only upregulated 4-fold after induction by glucose (Fig. S4D).

## DISCUSSION

Autotoxins and pathogens accumulated in the soil aggravate root rot diseases (4, 11). Previous studies have shown that some autotoxins inhibit the performance of the plant species that produce them and promote the growth and pathogenicity of soilborne pathogens (14). In this study, we found that the autotoxic ginsenoside Rg_1_ could cause root cell death, releasing cell wall degradants, including cellobiose and d-galacturonic acid, which could aggravate root rot disease by reshaping the rhizosphere microbiome to be more conducive to the disease. In particular, they could promote the growth and infection of the soilborne pathogen I. destructans by upregulating pathogenicity-related genes.

Rg_1_ was found to significantly increase the contents of cell wall degradants, including those degrading cellulose, pectin, and hemicellulase, but to decrease root metabolites related to the ascorbic acid-glutathione cycle. These findings were consistent with those of our previous study that showed Rg_1_ could induce a burst of reactive oxygen species (ROS) in root cells and upregulate cell wall decomposition-related genes (22). Many studies have demonstrated that autotoxins can induce a burst of ROS to cause cell death (25, 26). Moreover, some allelopathic compounds (27), heavy metals (28), formaldehyde (29), and other industrial chemical pollutants (30) have also been shown to cause cell death by inducing excessive accumulation of ROS. Taken together, these findings indicate that stresses caused by exogenous abiotic substances can lead to a burst of ROS in roots, which might cause the death of cells, releasing cell wall degradants.

Rg_1_, as well as cellobiose and d-galacturonic acid, could aggravate the severity of root rot disease by modifying the rhizospheric microbiome. Rhizodeposits, some of which act as carbon sources, signals, or antimicrobial substances, play an important role in modifying the rhizosphere microbiome (31, 32). Exogenous addition of these three substances into conditioned soil and natural soil aggravated the severity of root rot disease and drove changes in the soil microbiome in a similar direction. They could enrich some microorganisms that were positively related to root rot disease while suppressing negatively related ones. Importantly, some fungi positively related to the disease, such as *Ilyonectria*, have previously been reported as plant pathogens (23, 33). In soil experiments, the root rot pathogen I. destructans was frequently isolated from roots with typical root rot symptoms. In addition, the enrichment of the *Ilyonectria* species in the rhizospheric soil, as well as the positive correlation between its relative abundance and the severity of root rot disease, further confirmed that the occurrence of root rot disease was mainly because of buildup of I. destructans. Meanwhile, some fungi or bacteria negatively related to the disease, such as *Bradyrhizobium* (34), *Actinospica* (35), *Nocardia* (36), *Actinomadura* (37), Pseudomonas (38), and *Streptomyces* (39), have been reported as beneficial microorganisms for plant health. Soil inoculation experiments using culturable bacterial isolates confirmed that isolates positively related to the disease inhibited the emergence of seedlings and aggravated the severity of root rot disease, while isolates negatively related to the disease alleviated the adverse effects caused by positively related isolates. Therefore, Rg_1_, cellobiose, and d-galacturonic acid could drive the rhizosphere microbiome toward one that was conducive to root rot disease. Cellobiose showed the strongest ability to aggravate root rot disease, followed by Rg_1_ and then d-galacturonic acid. This may be because of the different strengths with which these substances modify the structures of the rhizosphere microbiome. Correlation analysis data confirmed that cellobiose could regulate the largest number of root rot disease-related microorganisms, followed by Rg_1_ and then d-galacturonic acid.

Besides the roles of Rg_1_, cellobiose, and d-galacturonic acid as carbon sources that drive change in the rhizosphere microbiome, antagonism between microorganisms positively and negatively related to the disease may be involved in microbiome modification. For example, bacteria from the *Streptomycetes* and Pseudomonas genera, which were negatively correlated with root rot disease, were frequently reported as plant growth-promoting rhizobacteria (PGPR) that promote plant growth (40, 41), induce plant disease resistance (42), occupy various niches of the rhizosphere (43), promote nutrition utilization (44), and antagonize pathogens (45). An *in vitro* antagonistic activity test confirmed that isolates from the Pseudomonas and *Streptomyces* genera, which were negatively correlated with the disease, exerted significant antagonistic activities against I. destructans. In addition, the Pseudomonas isolate also showed obvious antagonistic activity against bacterial isolates from the *Microbacterium* and *Ramlibacter* genera, which were positively correlated with the disease. These findings corroborate the knowledge that interactions among soil microorganisms play an important role in complex microbiomes (46). However, the microorganism interactions are likely very complex, which can be influenced by diverse factors, including host genotypes (47), niche specificities (43), and spatial distributions (48), and will be the subject of future studies.

Rg_1_, cellobiose, and d-galacturonic acid could promote infection by soilborne pathogens by upregulating pathogenicity-related genes. Many autotoxins, including cinnamic acid, ginsenoside, and ferulic acid, have been reported to promote the growth or pathogenicity of pathogens (25, 49, 50). Here, in addition to the autotoxin Rg_1_, the cell wall degradants cellobiose and d-galacturonic acid exerted synergistic effects with the soilborne pathogen I. destructans to aggravate root rot disease in hydroponic and sterilized-soil experiments. Further studies found that Rg_1_, cellobiose, and d-galacturonic acid could significantly promote the hyphal growth and spore germination of I. destructans; however, the underlying mechanisms employed by these three substances were different. Transcriptome analysis demonstrated that Rg_1_ promoted the growth and pathogenicity of I. destructans by upregulating the expression of mitosis, toxin synthesis, and CAZyme-related genes. Upregulated CAZyme genes may be involved in the utilization of Rg_1_ for growth. Previous reports demonstrated that ginsenosides could be hydrolyzed by microbial glycosyl hydrolases to release glycosyl as a nutrient for microorganisms (51). We also confirmed that Rg_1_ could be used as the sole carbon source by I. destructans. However, cellobiose and d-galacturonic acid primarily upregulated cell wall-degrading enzyme (CWDE) genes. Necrotrophic fungi can secrete large amounts of CWDEs to degrade cell wall polymers (52). Previous studies demonstrated that cellobiose and d-galacturonic acid could induce the synthesis of cellulase and pectinase, respectively (53). In the present study, we confirmed that cellobiose could significantly upregulate the expression of cellulase and hemicellulase genes, and d-galacturonic acid could significantly upregulate the expression of pectinase genes (54). Moreover, other Rg_1_-induced cell wall degradants, such as sophorose and gentiobiose, were also reported as inducers of the secretion of fungal CWDEs (55, 56). Overall, these findings indicate that autotoxins and many root cell wall degradants induced by autotoxins can promote the growth and infection of pathogens by upregulating pathogenicity-related genes. Cellobiose could induce higher levels of expression of genes involved in CAZymes of I. destructans, which implied that cellobiose had a high ability to enhance the growth and pathogenicity of I. destructans, resulting in its enrichment in soil. However, whether other rhizospheric microorganisms that were positively correlated with the disease were enriched by these three substances with similar mechanisms remains to be further studied.

In conclusion, autotoxins significantly changed the root metabolites of the host plant. Both autotoxins and the cell wall degradants that they induced could aggravate root rot disease by reassembling the rhizosphere microbiome, resulting in the enrichment of pathogens and microorganisms positively related to the disease but suppression of beneficial microorganisms. In particular, they could enhance the infection of pathogens by upregulating pathogenicity-related genes. Deciphering this autotoxin-mediated mechanism among plants, their associated pathogens, and their microbiomes will advance our fundamental knowledge and ability to degrade autotoxins or employ the microbiome to alleviate root rot disease in agricultural systems.

## MATERIALS AND METHODS

### Root metabolomics affected by the autotoxin Rg_1_.

The ginsenoside Rg_1_ (purity of ≥98%; Guizhou Dida Biological Technology Co., Guiyang, China) was dissolved in methanol (Bodi Chemical Co., Tianjin, China) and then diluted in distilled water to a final concentration of 1.0 μg ml^−1^. Distilled water containing the same concentration of methanol (0.1%, vol/vol) was used as a control. The roots of 1-year-old seedlings of P. notoginseng were immersed in Rg_1_ solution or a control for 0, 3, 12, 24, or 48 h. The fibrous roots from seedlings at each time point were rapidly harvested, frozen in liquid nitrogen, and stored at −80°C until use. Root samples for metabolite profiling were obtained in six independent replicated experiments. The metabolites were extracted by grinding 100 mg of root tissue in 0.4 ml of chloroform/methanol (1:3) with ribitol (0.2 mg ml^−1^) added as an internal standard (IS). A 1-μl drop of each sample was injected into an Agilent 7890 gas chromatograph (Agilent, Santa Clara, CA, USA) coupled to a 5973 mass spectrometer at a split ratio of 1:1 using GC-TOF MS methodology. Separation was achieved using an Agilent DN-5MS column (30 m by 250 μm by 0.25 μm; J&W Scientific, Folsom, Santa Clara, CA, USA) at a flow rate of 1.0 ml min^−1^. The MS data were acquired in scan mode over a range of *m/z* 50 to 800 at a rate of 20 spectra/s. All GC/MS data were processed using the Leco ChromaTOF software (version 3.25). Peaks with signal-to-noise (S/N) ratios lower than 30 were rejected, after which the FiehnLib libraries and the NIST 2014 library were used for metabolite identification (57). Each peak area was then normalized according to the IS before multivariate data analysis was performed. The intensities of the identified peaks were integrally normalized against the sum of the peak intensities for each sample, followed by log transformation. Pairwise comparisons between the control and different time points were performed separately for each metabolomics platform using the same modeling approach. Normalized data were analyzed using the SIMCA-*P*+ program (version 13.0; Umetrics AB, Umeå, Sweden). The validated data matrix was analyzed using multivariate partial least-squares discriminant analysis (PLS-DA), followed by the identification of important metabolites using orthogonal partial least-squares discriminant analysis (OPLS-DA) supported by a regression coefficient plot and variable importance in the projection (VIP) values. A *P* value threshold of <0.05 and a VIP value of >1 were used to select potentially different metabolites. To test the validity of the results from metabolite profiling, 13 metabolites were randomly selected for quantification by GC-TOF MS, using comparison with standard compounds.

### Effects of Rg_1_, cellobiose, and d-galacturonic acid on root rot disease of *P. notoginseng*.

To assess the effects of Rg_1_, cellobiose, and d-galacturonic acid on root rot disease of P. notoginseng, we conducted experiments based on two types of soil: (i) natural soil collected from the surface (<15 cm) in a pine forest in Xundian County (25°30′35″N, 103°17′52″E; 1,876 m altitude), and (ii) conditioned soil prepared as previously described, with some modifications (58). Briefly, 95% sterilized natural soil (autoclaved at 121°C for 30 min) was amended with 5% soil in which P. notoginseng had been cultivated continuously for 3 years that was collected from farmland located in Xundian County (25°30′8″N, 103°11′18″E; 1,876 m altitude). Pots (17.5 cm in diameter at the top, 12.5 cm in diameter at the bottom, and 14 cm in height) were each filled with a total of 1.7 kg of one soil type, after which five 1-year-old seedlings were transplanted into each pot. Ginsenoside Rg_1_ was dissolved in methanol and cellobiose and d-galacturonic acid (Sigma-Aldrich Co. St. Louis, Missouri) were dissolved in sterilized deionized water to prepare stock solutions at final concentrations of 0, 0.01, 1, and 100 μg ml^−1^ that were subsequently watered into pots until saturation. There were 15 pots per treatment, using a completely randomized experimental design, and this experiment was repeated three times. During the experiment, the disease/survival ratio of seedlings was recorded every 4 (in conditioned soil experiment) or 7 (in natural soil experiment) days, after which seedlings were collected and disease index values were investigated as follows: disease index = 100 × ∑(disease level × seedling number)/total seedling number × highest disease level. Disease was distinguished into six levels, as follows: 0, no root rot; 1, the rotting area accounted for less than 5% of the root system; 2, the rotting area accounted for 6% to 10%; 3, the rotting area accounted for 11% to 25%; 4, the rotting area accounted for 26% to 50%; 5, the rotting area accounted for 51% to 75%; and 6, the rotting area accounted for 75% to complete decomposition. A total of 150 rhizosphere soil samples was collected for further analysis.

### Rhizosphere microbiome analysis.

Rhizospheric soil samples from conditioned soil and natural soil that had been treated with these three substances at concentrations of 0 and 0.01 μg ml^−1^ were selected for microbiome analysis by 16S rRNA and internal transcribed spacer (ITS) gene sequencing. Genomic DNA was extracted using the PowerSoil DNA isolation kit (Mo Bio/Qiagen), following the instruction manual. DNA quantity and quality were determined using a NanoDrop 2000 spectrophotometer (Thermo Scientific, USA). The DNA was used in the study only when the *A*_260_/*A*_280_ ratio was larger than 1.7 and the *A*_260_/*A*_230_ ratio was larger than 1.8. The V4-V5 region of the 16S rRNA gene for bacteria was amplified with the universal primers F515 (5′-GTGCCAGCMGCCGCGG-3′) and R907 (5′-CCGTCAATTCMTTTRAGTTT-3′) (59, 60). The fungal ITS2 gene was amplified with the universal primers ITS3-2024F (5′-GCATCGATGAAGAACGCAGC-3′) and ITS4-2409R (5′-TCCTCCGCTTATTGATATGC-3′) (61). A total of 6.46 Gb of raw reads was obtained after sequencing using an Illumina HiSeq 2500 sequencer (Illumina, USA), after which sequences were assembled according to a default script in QIIME (version 1.9.1) (62). Through quality filtering and chimera removal (63), effective sequences were generated and then used to identify operational taxonomic unit (OTU) clusters based on 97% pairwise identity using the UPARSE software (version 7.0.1090) (64). Taxonomic assignment of each OTU was then performed against the Unite database (version 7.0) and the Silva database (version 128) for fungi and bacteria, respectively (65). The data of each sample were processed by normalization based on the minimum data in the sample. The alpha diversity indices (Chao1, Sobs, Ace, Shannon, and Simpson indices) were calculated using Mothur (version 1.34.4) (66). Then, the relative abundances of individual OTUs were calculated and used for further statistical analyses.

### Isolation, identification, and function analysis of modified soil bacteria in root rot disease.

Rhizosphere bacteria were isolated using 1/10 tryptic soy agar (TSA) and R2A medium by gradient dilutions as previously described (67). All purified isolates were identified with universal primers 27F (AGAGTTTGATCCTGGCTCAG) and 1492R (TACGGCTACCTTGTTACGACTT) and then stored in lysogeny broth at −20°C.

To determine whether the function of the isolates was significantly correlated with root rot severity, one isolate each from the *Microbacterium*, *Variovorax*, and *Ramlibacter* genera (positively disease-related genera) and from the Pseudomonas, *Streptomyces*, and *Pseudarthrobacter* genera (negatively disease-related genera) was selected randomly for further study. First, sterilized soil was placed in pots and planted with five 1-year-old seedlings per pot, followed by watering with 200 ml of a water suspension of a single isolate or a combination of one positively disease-related and one negatively disease-related isolate at a final concentration of 10^6^ CFU ml^−1^. Each treatment had five replicates, and the experiment was performed three times. Two months later, the emergence rate of P. notoginseng seedlings was measured. Four months later, root rot disease index values were investigated as described above. Second, the antagonistic activities of the above-mentioned isolates against Ilyonectria destructans were tested as previously described, with some modifications (68). Briefly, a mycelium block (5 mm in diameter) of I. destructans was placed in the middle of a potato dextrose agar (PDA) plate. Next, the culturable bacterial isolates were placed at equal distances (30 mm) around the pathogen mycelium block. A plate with only the mycelium block of I. destructans grown on PDA was used as the control. All treatments were incubated at 25°C for 7 days. The mycelium growth of the pathogen was determined by measuring the colony semidiameter. Each treatment had four replicates, and the experiment was performed three times. Third, pairwise interaction assays of the aforementioned bacterial isolates were conducted using the Burkholder plate assay to test the inhibition between the isolates. Broth cultures (100 μl) of the six strains growing in nutrient agar (NA) at 25°C for 2 days were spread onto NA medium to create bacterial lawns, after which 10 μl of each broth culture was transferred onto the lawns, resulting in a total of 36 interactions (6 × 6), six of which were self-inhibition controls. Plates were placed at 25°C, and inhibition was scored as the presence/absence of clearing bacteriostatic zones after 3 days of incubation.

### Effects of Rg_1_, cellobiose, and d-galacturonic acid on the pathogenicity of *I. destructans* under hydroponic and sterilized-soil conditions.

The effects of Rg_1_, cellobiose, and d-galacturonic acid on the pathogenicity of I. destructans in hydroponics were evaluated as previously described, with some modifications (22). Briefly, batches of five 1-year-old seedlings were grown hydroponically in tissue culture bottles (200 ml) that were filled with 80 ml sterilized deionized water. Next, Rg_1_, cellobiose, and d-galacturonic acid were exogenously added at final concentrations of 0, 0.01, 1, and 100 μg ml^−1^ with or without conidial spores (10^3^ conidia ml^−1^). Each treatment had four replicates, and the experiment was repeated three times. All bottles were arranged in the same chamber in a completely randomized block design and incubated under a light/dark cycle of 16 h/8 h at 25 ± 1°C and a relative humidity of 80% to 95%. Fresh weights of seedlings were recorded at the beginning and the end of the experiment to monitor wilting.

To assess the effects of Rg_1_, cellobiose, and d-galacturonic acid on the pathogenicity of I. destructans in sterilized soil, six 1-year-old seedlings were transplanted into each pot, after which they were watered with the ginsenoside Rg_1_ and the root degradants cellobiose and d-galacturonic acid at final concentrations of 0, 0.01, 1, and 100 μg ml^−1^, with or without conidial spores at a final concentration of 10^3^ conidia ml^−1^, until saturation. All pots were arranged in the same greenhouse in a completely randomized block design. Each treatment was repeated for five pots, and this experiment was performed three times. After 26 days, seedlings of P. notoginseng were collected to measure the root rot index values as described as above.

The effects of Rg_1_, cellobiose, and d-galacturonic acid on the spore germination and mycelium growth of I. destructans were tested as previously described, with some modifications (51). Briefly, PDA plates amended with Rg_1_, cellobiose, and d-galacturonic acid at concentrations of 0, 0.01, 0.1, 1, 10, and 100 μg ml^−1^ were prepared. The final concentration of methanol in the medium amended with Rg_1_ and its control was limited to 0.1% (vol/vol). For spore germination, 150 μl (10^6^ conidia ml^−1^) of conidial suspension was spread onto plates with amended PDA medium. After 6 h of incubation in the dark at 25°C, conidial germination was observed in five randomly selected microscopic fields for each amended medium using a 20× Leica microscope (Leica DM 2000). The germination rate was expressed as the percentage of germinated spores relative to the total spores calculated. For mycelium growth, the inocula (5 mm in diameter) of I. destructans growing on PDA plates were transferred onto amended PDA plates and incubated in the dark at 25°C until the colony diameters reached two-third of the plates. The diameters of the colonies were measured. These two experiments were performed three times with four replicates in each treatment.

### Effects of Rg_1_, cellobiose, and d-galacturonic acid on the transcriptome of *I. destructans*.

A total of 1 ml of conidial spores (10^6^ conidia ml^−1^) was preincubated in 300 ml of potato dextrose liquid medium in a 1-liter culture flask on an orbital shaker (ZHWY-111B; Shanghai Zhicheng Analytical Instruments Manufacturing Co., Ltd.) at 25 ± 1°C and 120 rpm. After 72 h, samples were divided into six equal aliquots (50 ml each in 200-ml culture flasks), three of which were added with Rg_1_ stock solution to a final concentration of 1 μg ml^−1^ and the other three added with methanol to a final concentration of 0.1% (vol/vol) as a control. Mycelia were collected after 6 h of exposure to Rg_1_ (incubation at 25 ± 1°C and 120 rpm on an orbital shaker) by centrifugation at 4°C and 10,000 × *g* (centrifuge 5804 R; Eppendorf, Hamburg, Germany) for 5 min. Samples were then immediately frozen in liquid nitrogen and stored at −80°C.

To assess the effects of cellobiose and d-galacturonic acid on the transcriptome of I. destructans, preincubation was performed as described above, after which inducing progression was performed as described by Cooper and Wood (69), with slight modifications. Briefly, preincubated mycelia were centrifuged (8,000 × *g* and 4°C), washed three times with sterilized deionized water, and then resuspended in 450 ml of salt medium (1 g liter^−1^ KH_2_PO_4_, 0.5 g liter^−1^ MgSO_4_·7H_2_O, 4.6 g liter^−1^ Casamino acids, 0.2 mg liter^−1^ FeSO_4_·7H_2_O, 1.0 mg liter^−1^ ZnSO_4_·7H_2_O, 0.02 mg liter^−1^ NaMoO_4_·2H_2_O, 0.02 mg liter^−1^ CuSO_4_·5H_2_O, 0.02 mg liter^−1^ MnCl_2_·4H_2_O, pH adjusted to 5.5 with NaOH). After 12 h of incubation at 25 ± 1°C and 120 rpm under conditions of starvation, samples were divided into nine aliquots (50 ml each in 200-ml culture flasks), three of which were induced with one carbon source. Capsules containing 0.05 g ml^−1^ glucose, cellobiose, or d-galacturonic acid were placed into each culture flask. The capsules were prepared in sterilized 2-ml centrifuge tubes covered with 4 layers of 25-kDa dialysis membrane (Spectra/Por 6, standard, RC, 25 kDa, 34 mm wide; Sangon Biotech, Co., Ltd. Shanghai, China) to release carbon sources evenly throughout the experiment. After 0 and 12 h of incubation at 25 ± 1°C and 120 rpm using an orbital shaker, mycelia were collected and stored as described above. This experiment was repeated three times.

Total RNA was extracted from mycelia using the TRIzol (Bio-Rad, USA) method, and mRNA with a poly(A) tail was enriched by oligo(dT) magnetic beads (Invitrogen, USA). Because no reference genome was available for I. destructans, a transcriptome assembly library was constructed as a reference library by mixing equal amounts of RNA from the above-described 18 samples. The libraries were sequenced on the BGISEQ-500 platform by BGI (Shengzhen, China). Clean reads were assembled *de novo* using the Trinity program (version 2.0.6). Unigenes were then annotated employing the NR, NT, Swiss-Prot, PHI, KEGG, Gene Ontology (GO), and Pfam databases. Gene expression analysis was performed in two sequential steps. First, all clean reads were mapped to the assembled sequences using Bowtie 2 (version 2.2.5) to calculate the read counts for each transcript (70). The transcript abundance for each gene was then measured and normalized as fragments per kilobase of exon per million fragments mapped (FPKM) values (71). To test the validity of the results from the transcriptome, three differentially expressed genes were randomly selected for quantification by RT-qPCR. The primers are listed in Table S1.

### Data analysis.

SPSS software (version 18.0) was used for general statistical analyses. Normality of distribution and homogeneity of variance were checked before statistical analysis. The mean separations among treatments were analyzed by one-way analysis of variance (ANOVA) and Duncan’s multiple-range test (*P < *0.05). For transcriptomics analysis, differentially expressed genes (DEGs) were functionally classified according to the GO annotation results and official classification, the phyper function in R software (version 3.5.1) was used to conduct enrichment analysis, false discovery rate (FDR) corrections were made to *P* values, and a *q* value of ≤0.05 was considered to show significant enrichment. Heatmaps were used to show the DEGs, which were selected as | log_2_ fold change (FC) | > 2 with the average FPKM ranked in the top 50 and then calculated as log_10_(FPKM + 1). The results of the cluster analysis of gene expression were evaluated using heatmap.2 in the gplots package in R. It should be noted that DEGs in cellobiose and d-galacturonic acid treatments were put together and selected as described above. Pearson’s correlation coefficient within the R software was employed to correlate the root rot severity and relative abundance of microorganisms at the genus level, with | *r* | > 0.6 and *P < *0.05 considered to show significant correlation. The correlation analysis was visualized using Cytoscape (version 3.6.1).

### Data availability.

The clean ITS and 16S rRNA sequences reported in this article have been deposited in the NCBI Short Read Archive (SRA) under accession number PRJNA601797. The accession number for RNAseq clean data is PRJNA602217.
