# Supplementary material for: Autotoxin Rg1 Induces Degradation of Root Cell Walls and Aggravates Root Rot by Modifying the Rhizospheric Microbiome
Source: Microbiol Spectr. 2021 Dec 15;9(3):e01679-21. doi: 10.1128/spectrum.01679-21 (PMC8672892; doi:10.1128/spectrum.01679-21)
Supplement: SUPPLEMENTAL FILE 1 — Supplemental material. Download Spectrum01679-21_Supplemental_Material.pdf, PDF file, 1.1 MB [file spectrum01679-21_supplemental_material.pdf]

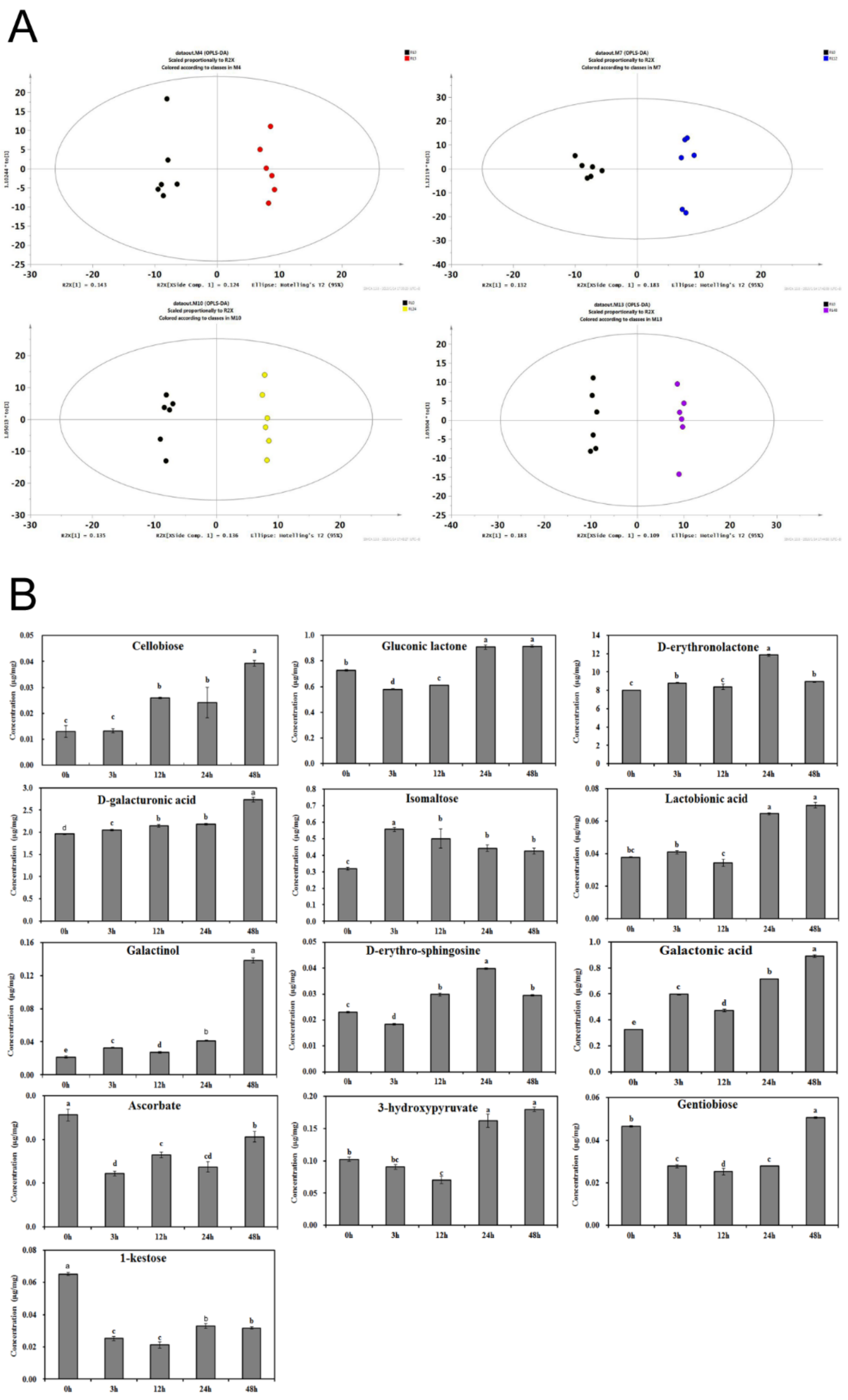

Figure S1 (A), Score plots of OPLS-DA model obtained from 0 h and 3 h, 0 h and 12 h, 0 h and 24 h and 0 h and 48 h; (B), Levels of the 13 metabolites monitored by GC-TOF-MS combined with standard compounds in roots exposed to Rg_1 for different time points. Data were obtained for six independent biological replicates. The values are the means ± SE. Bars with different letters are significantly different ($p < 0.05$; $n = 6$).

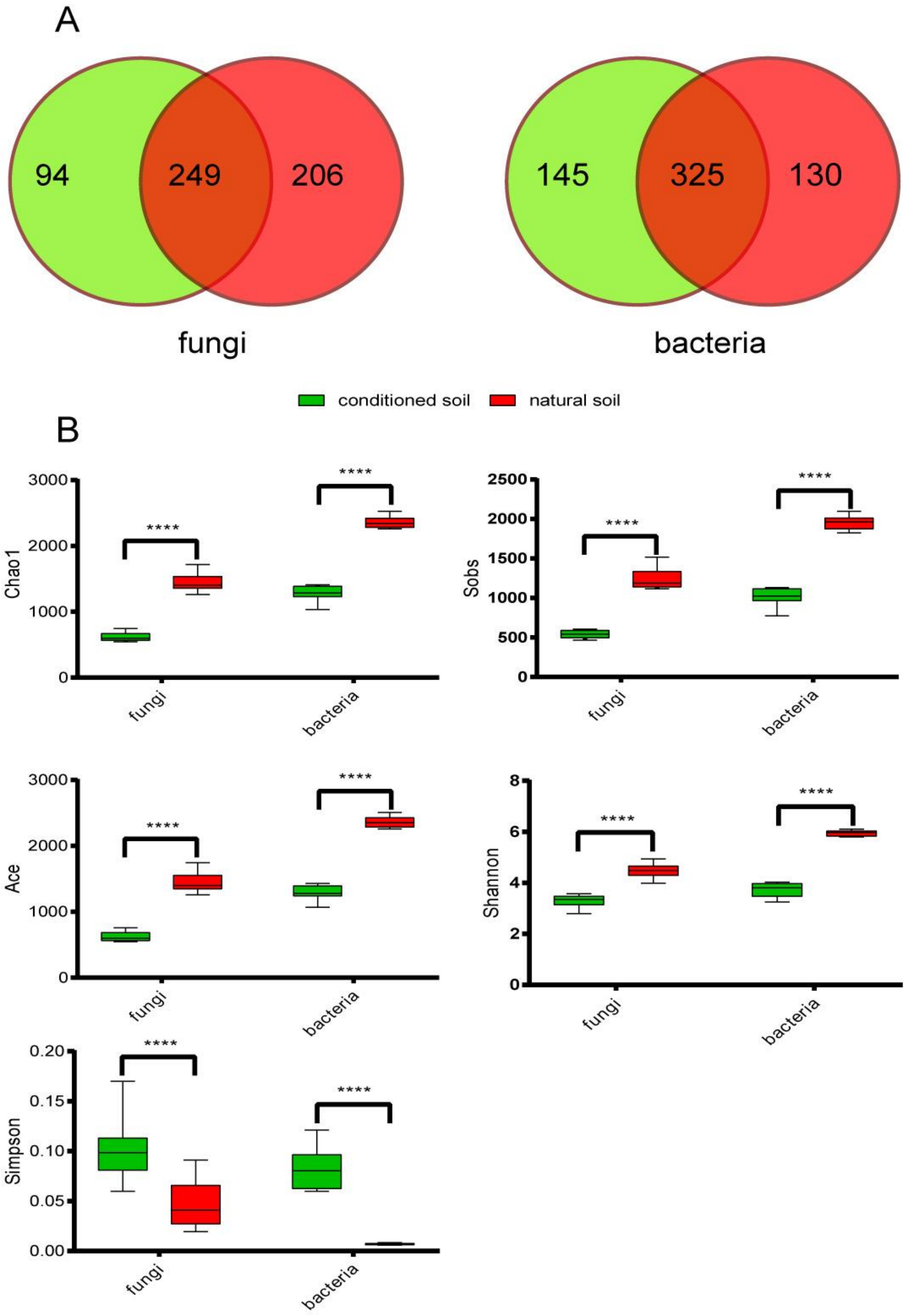

Figure S2 (A), Number of taxa of genera of fungi and bacteria in the rhizosphere of *P. notoginseng* in conditioned soil and natural soil detected by ITS or 16S rRNA sequencing; (B), alpha diversity comparison between conditioned soil and natural soil. The significance between two types of soils were determined by multiple t tests and corrected for multiple comparisons using the Holm-Sidak method, **** means p <0.0001.

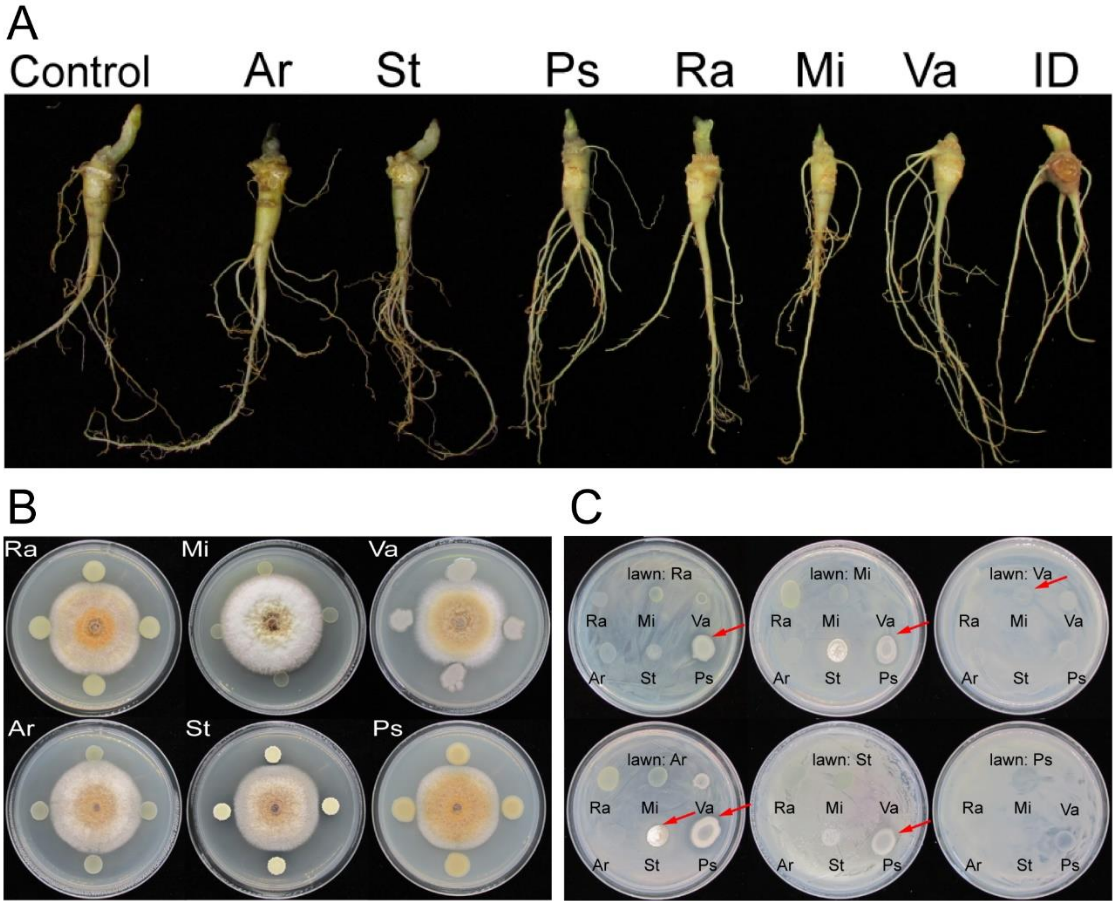

Figure S3 (A), Pathogenic tests of six culturable bacterial isolates and pathogenic fungi *Ilyonectria destructans* to *Panax notoginseng*; (B), antagonistic activities of the six culturable bacterial isolates against *I. destructans*; (C), antagonistic activities of the six culturable bacterial isolates among each other. Ar, *Pseudarthrobacter*; St, *Streptomyces*; Ps, *Pseudomonas*; Ra, *Ramlibacter*; Mi, *Microbacterium*; Va, *Variovorax*; Control and ID in (A) respectively means no inoculation and *I. destructans* inoculation. The red arrow in (C) indicates the bacteriostatic zones.

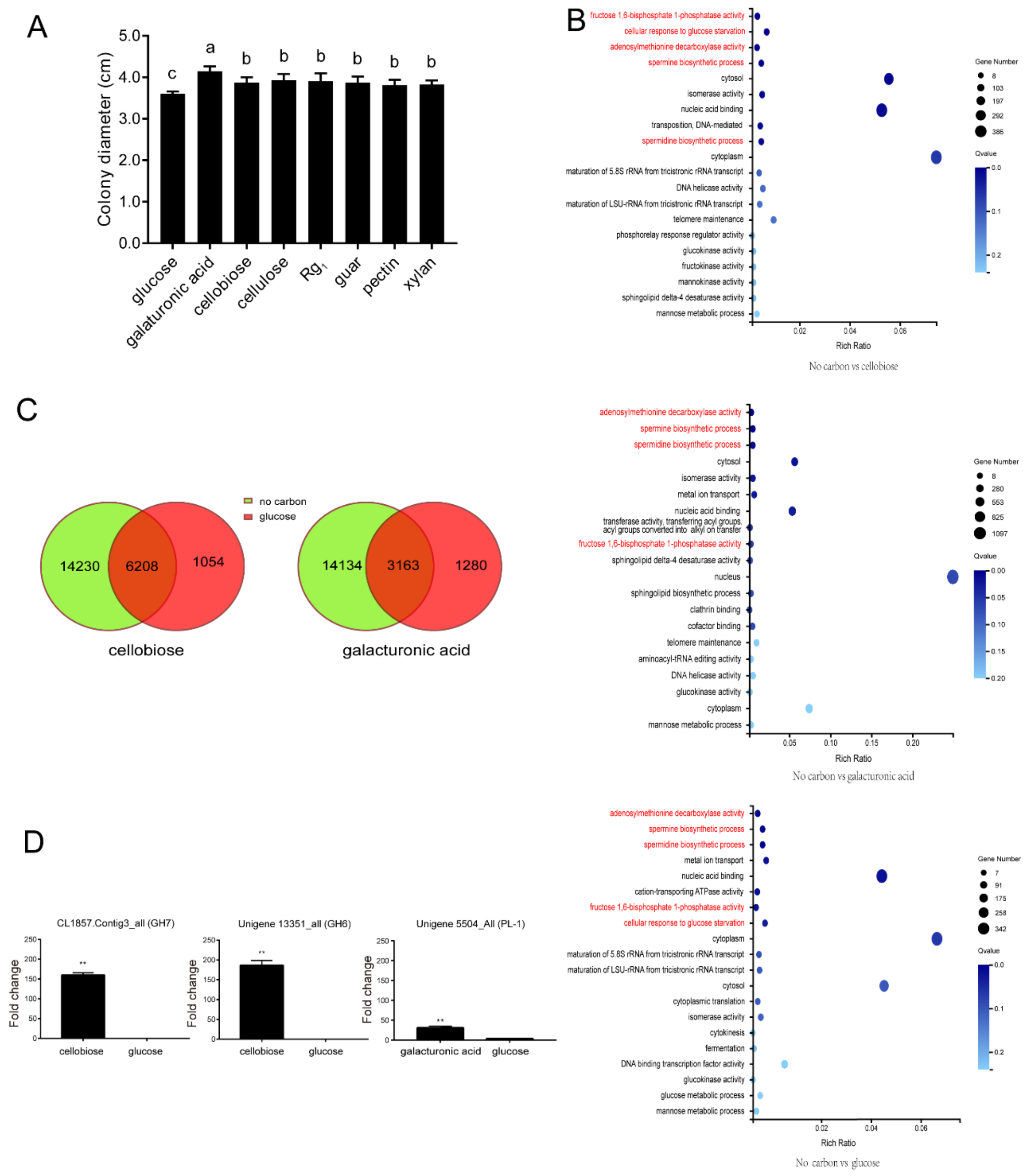

Figure S4 (A), Colony diameters of *Ilyonectria destructans* on medium containing glucose as the sole carbon source, as well as D-galactronic acid, cellobiose, cellulose, Rg_1, guar, pectin and xylan; (B), GO enrichment analysis of *I. destructans* when there was no carbon source compared with the presence of cellobiose, D-galacturonic acid or glucose as the sole carbon source; (C), Number of DEGs in cellobiose and D-galacturonic acid treatment compared with both negative control (no carbon source) and positive control (glucose as carbon source); (D), RT-qPCR confirmation of DEGs.

Table S1 Primers used for RT-qPCR

Gene ID	Forward primer	Reverse primer
Unigene13351_All (GH6)	5'-CTCGGTTGCCGTTTCTACCA-3'	5'-TGACGCCGGAATAAGGGTTG-3'
CL1857.Contig3_All (GH7)	5'-AAGGGCCAATACAGCACCAA-3'	5'-GCCACAGCTGATACCCGAAA-3'
Unigene5504_All (PL-1)	5'-CTCTTCTCACCGCTCTCCTG-3'	5'-GGTCTGGGCAGACTTGTAGG-3'

Table S2 The significantly changed sanqi root metabolites at different time length after exposure to Rg_1

Metabolites	Fold change			
	3h	12h	24h	48h
cellobiose	139.5	1812.2	1803.9	1580.2
isomaltose	2.4	2.8	4.8	5.3
2-deoxy-D-glucose	1.6	2.6	0.8	0.4
fructose-6-phosphate	1.0	1.3	1.2	1.5
digitoxose	1.9	2.1	1.5	1.7
sorbose	5.4	4.5	8.3	6.3
sophorose	2.4	3.9	4.5	3.8
methyl-beta-D-galactopyranoside	1.0	1.9	2.2	1.6
D-glucoheptose	1.8	7.7	6.1	1.3
lyxose	1.4	4.3	5.8	8.0
galactinol	1.2	1.1	552.0	1668.2
gentiobiose	-408.3	-1.5	-1.8	1.0
lactose	-5.4	-1.3	-2.5	1.3
2-deoxy-D-galactose	-2.9	-3.8	1.3	1.3
1-kestose	-1.4	-1.5	-3.9	-2821.5
raffinose	-1.5	-1.4	-2.1	-3.4
D-galacturonic acid	298.5	396.0	444.4	445.6
lactobionic acid	1.2	265.0	1381.3	366.9
3-hydroxypyruvate	-869.9	-2.9	-1.3	1.2
1-aminocyclopropanecarboxylic acid	-1.7	-1.7	-2.4	-98.6
glutamic acid	1.6	1.8	1.9	2.2
ornithine	1.3	1.9	3.5	1.8
aspartic acid	1.5	-1.3	1.0	2.1
tryptophan	1.0	1.1	1.5	1.5

threo-beta-hyrdoxyaspartate	-1.3	3.1	2.2	5.4
adenosine 5-monophosphate	-5.1	1.2	-1.1	-1.3
leucine	1.3	-1.2	-2.6	-23990.6
D-erythro-sphingosine	1.2	1.1	899.4	1445.7
1,3-diaminopropane	1.1	1.3	1.6	2.7
gluconic lactone	1225.6	17027.8	19069.2	33862.2
D-erythronolactone	1.2	733.3	373.6	266.2
1-methyladenosine	1.4	2.2	1.9	2.4
prunin degr. Prod.	1.2	1.1	65.9	214.9
ascorbate	-178.6	-5.3	-1.5	-2.0

Table S3 Culturable bacterial isolates obtained from rhizospheric soil

isolated genera	successive soil	natural soil	the number of isolates
Agrococcus	+	-	5
Bacillus	+	+	32
Bosea	+	+	10
Brevundimonas	+	+	1
Chryseobacterium	+	+	1
Curtobacterium	+	-	1
Devosia	+	+	3
Dyadobacter	+	+	2
Fictibacillus	+	-	4
Flavihumibacter	+	+	2
Lentzea	+	+	1
Lysinibacillus	+	+	1
Lysobacter	+	+	18
Massilia	+	+	1
Mesorhizobium	+	+	1
Microbacterium	+	+	46
Nocardioides	+	+	6
Novosphingobium	+	+	1
Paenibacillus	+	+	1
Pedobacter	+	+	1
Phyllobacterium	+	-	1
Planomicrobium	+	+	1
Pseudarthrobacter	+	+	44
Pseudomonas	+	+	8
Pseudoxanthomonas	+	+	10
Ramlibacter	+	+	3

Rhizobium	+	+	2
Rhodanobacter	+	+	1
Rhodobacter	+	+	5
Salana	+		1
Sphingobium	+	+	2
Sphingomonas	+	+	4
Sphingopyxis	+	+	40
Sphingosinicella	+	-	1
Sporosarcina	+	+	4
Streptomyces	+	+	10
Thermomonas	+	+	1
Variovorax	+	+	3

"+" means this bacterial genera was detected in successive and/or natural soil through ITS and 16SrRNA sequencing.

Table S4 GO enriched DEGs associated with G protein pathway after exposure to Rg_1 for 6 h

Gene ID	KEGG Orthology	log2(Rg_1/Control)	Q value
Unigene21789_All	K04630//guanine nucleotide-binding protein G(i) subunit alpha	9.28	$4.14E^{-57}$
Unigene12130_All	K04630//guanine nucleotide-binding protein G(i) subunit alpha	8.13	$3.40E^{-30}$
CL357.Contig5_All	K04630//guanine nucleotide-binding protein G(i) subunit alpha	4.87	$1.13E^{-17}$
Unigene12110_All	K04630//guanine nucleotide-binding protein G(i) subunit alpha	2.49	$3.07E^{-52}$
Unigene12121_All	K04630//guanine nucleotide-binding protein G(i) subunit alpha	2.39	$2.08E^{-130}$
CL5271.Contig4_All	K04630//guanine nucleotide-binding protein G(i) subunit alpha	2.23	$5.40E^{-10}$
CL5271.Contig6_All	K04630//guanine nucleotide-binding protein G(i) subunit alpha	1.99	0.00022
Unigene12136_All	K04630//guanine nucleotide-binding protein G(i) subunit alpha	1.18	$8.61E^{-79}$
Unigene21790_All	K04630//guanine nucleotide-binding protein G(i) subunit alpha	-1.16	$9.41E^{-16}$
CL357.Contig4_All	K04630//guanine nucleotide-binding protein G(i) subunit alpha	-1.17	$5.21E^{-24}$
Unigene12123_All	K04630//guanine nucleotide-binding protein G(i) subunit alpha	-1.17	$6.37E^{-06}$
Unigene21792_All	K04630//guanine nucleotide-binding protein G(i) subunit alpha	-1.177	$4.97E^{-06}$
CL3592.Contig2_All	K04630//guanine nucleotide-binding protein G(i) subunit alpha	-1.23	$2.12E^{-30}$
CL5271.Contig5_All	K04630//guanine nucleotide-binding protein G(i) subunit alpha	-1.351721098	$9.49E^{-21}$
Unigene12114_All	K04630//guanine nucleotide-binding protein G(i) subunit alpha	-1.828449985	$1.49E^{-48}$

Unigene12107_All	K04630//guanine nucleotide-binding protein G(i) subunit alpha	-1.98	$4.61E^{-12}$
Unigene12118_All	K04630//guanine nucleotide-binding protein G(i) subunit alpha	-2.28	$8.24E^{-06}$
Unigene12126_All	K04630//guanine nucleotide-binding protein G(i) subunit alpha	-3.27	$6.89E^{-11}$
Unigene12113_All	K04630//guanine nucleotide-binding protein G(i) subunit alpha	-3.57	$6.65E^{-21}$
Unigene12125_All	K04630//guanine nucleotide-binding protein G(i) subunit alpha	-4.43	$4.98E^{-08}$
Unigene12116_All	K04630//guanine nucleotide-binding protein G(i) subunit alpha	-5.06	$1.84E^{-05}$
CL357.Contig3_All	K04630//guanine nucleotide-binding protein G(i) subunit alpha	-5.22	$5.90E^{-06}$
Unigene12112_All	K04630//guanine nucleotide-binding protein G(i) subunit alpha	-5.84	$2.08E^{-08}$
Unigene12119_All	K04630//guanine nucleotide-binding protein G(i) subunit alpha	-6.97	$2.21E^{-88}$
Unigene12137_All	K04630//guanine nucleotide-binding protein G(i) subunit alpha	-9.09	$2.32E^{-52}$
Unigene12412_All	K05857//phosphatidylinositol phospholipase C, delta [EC:3.1.4.11]+ko01100//Metabolic pathways+ko04933//AGE-RAGE signaling pathway in diabetic complications+ko04070//Phosphatidylinositol signaling system+ko00562//Inositol phosphate metabolism	-1.25	$1.40E^{-80}$
CL2874.Contig2_All	K07973//guanine nucleotide-binding protein subunit gamma, other+ko04011//MAPK signaling pathway - yeast	-1.15	$7.21E^{-44}$
CL2874.Contig1_All	K07973//guanine nucleotide-binding protein subunit gamma, other+ko04011//MAPK signaling pathway - yeast	-5.22	$6.14E^{-06}$

CL3596.Contig2_All	K11232//osomolarity two-component system, phosphorelay intermediate protein YPD1+ko04011//MAPK signaling pathway - yeast	-1.19	$2.85E^{-11}$
CL5180.Contig1_All	K19475//WAS/WASL-interacting protein+ko04144//Endocytosis	-2.73	$5.60E^{-21}$
